# Effects of physical exercise on mindfulness level among female college students: the mediating effect of mobile phone addiction

**DOI:** 10.3389/fpsyg.2025.1667868

**Published:** 2025-09-12

**Authors:** JiaYi Li, YiKun Zheng, LiHan Lin, XiuQi Li, GuoPeng Hu

**Affiliations:** ^1^College of Physical Education, Huaqiao University, Quanzhou, China; ^2^Provincial University Key Laboratory of Sport and Health Science, School of Physical Education and Sport Science, Fujian Normal University, Fuzhou, China; ^3^College of Foreign Languages, Huaqiao University, Quanzhou, China

**Keywords:** physical activity, female college students, mobile phone addiction, mindfulness, women’s physical activity promotion

## Abstract

**Background:**

This study explores the impact of physical exercise on female college students’ trait mindfulness levels and examines the mediating role of mobile phone addiction in this relationship.

**Methods:**

The empirical study employs stratified random cluster sampling. The Physical Activity Rating Scale-3 (PARS-3), Mindful Awareness Attention Scale (MAAS), and Mobile Phone Addiction Tendency Scale (MPATS) were used to conduct a questionnaire survey. The participants of the study were 554 female college students from 5 universities in Fujian Province, China.

**Results:**

The scores for physical exercise, trait mindfulness, and mobile phone addiction among female college students were 16.04 ± 15.92, 53.79 ± 11.45, and 47.93 ± 9.76, respectively. Physical exercise and trait mindfulness had a significant positive correlation (r = 0.20, *p* < 0.01), while physical exercise and mobile phone addiction had a significant negative correlation (r = −0.17, *p* < 0.01). Also, a significant negative correlation was found between mobile phone addiction and trait mindfulness (r = −0.45, *p* < 0.01). The structural equation model analysis showed that the direct effect of physical exercise on trait mindfulness was 0.071 (*p* < 0.01), and mobile phone addiction played a significant indirect mediating role in it, with the indirect effect being 0.044, accounting for 35.77% of the total effect.

**Conclusion:**

The findings indicate that physical exercise can enhance female college students’ trait mindfulness directly and indirectly by reducing mobile phone addiction. This implies the importance of promoting physical exercise and managing mobile phone use in universities, while combining mindfulness training with physical exercise may greatly benefit their comprehensive mental development. The cross-sectional design of this study can only reflect the co-occurrence of variables and cannot determine the causal direction. Using self-report scales, while accounting for potential social expectation biases and recall biases, future studies can further validate the conclusions through longitudinal tracking and multi-dimensional assessment.

## 1 Introduction

In recent years, social change has led to an increase in individualism, with traditional collectivist values gradually being marginalized (Kessler et al., 2007). This shift has exposed individuals to more stress and anxiety in their pursuit of self-realization. Coupled with environmental uncertainty, this has created a complex psychological stress structure, leading to a general increase in negative emotions. Female college students, in particular, are vulnerable to a range of emotional issues, including sleep disorders, neurasthenia, interpersonal sensitivity, depression, and anxiety (Lemola et al., 2015; Eisenberg et al., 2009). Furthermore, the high penetration of digital life, such as the irreplaceable role of mobile applications in learning and social interactions, as well as the implicit demands for “instant response” in social culture, may prompt individuals to frequently check their phones in order to maintain interpersonal relationships, thereby strengthening the daily reliance on mobile devices and indirectly increasing the risk of addiction (Aernout et al., 2021). According to the 52nd Statistical Report on Internet Development in China released by the China Internet Network Information Center (CNNIC), as of June 2023, the number of internet users in China reached 1.079 billion, with the average weekly internet usage time being 29.1 h, and 99.8% of users accessed the internet via smartphones (Zhao and Kuang, 2025). With the continuous development of a digital lifestyle, smartphones and the internet have gradually become core parts of the daily life of female college students. This not only changes their lifestyle but also affects their mental health (Joshi et al., 2023). Smartphone addiction not only triggers anxiety (Kwak et al., 2022), weakens self-control, leads to distraction (Zhang et al., 2023), and reduces sleep quality (Xie et al., 2023), but also causes insecurity and avoidance of real-life social interactions (Chen et al., 2023; Zhang et al., 2022). Additionally, prolonged immersion in smartphones may cause young people to lose their life goals and sense of self-worth, thereby lacking motivation to pursue the meaning of life (Wacks and Weinstein, 2021).

Mindfulness, originating from Buddhism, has weakened religious connotations in modern contexts and mainly refers to the awareness and acceptance of present experiences, encompassing three aspects: awareness, attention, and memory. In modern times, the religious connotations of mindfulness have been weakened, and it is mainly defined in descriptive and operational terms (Li et al., 2024). The descriptive definition emphasizes “acceptance” and “non-judgment,” while the operational definition views mindfulness as a trait state, consisting of “self-control of attention” and “orientation toward personal experience.” Among the various forms of mindfulness, trait mindfulness is a relatively stable personality trait, distinct from the transient state of mindfulness. It emphasizes an individual’s long-term ability to self-regulate attention and focus on personal experiences (Baer, 2003). In contemporary society, the cultural atmosphere that emphasizes efficiency and immediate gratification has profoundly influenced people’s thinking patterns and behavioral habits. The fast-paced lifestyle keeps people in a state of high-speed operation for a long time, and the popularity of smartphones and social media has continuously flooded the brain with a large amount of fragmented information, causing it to remain in an overly excited state and making it difficult to maintain deep concentration. At the same time, the intense social competition environment prompts people to constantly direct their attention towards future goals or external evaluation systems rather than the current internal experience. These factors jointly form a cognitive tendency that is outwardly demanding rather than inwardly perceptive, objectively weakening the core qualities such as concentration, self-awareness, and acceptance that are necessary for developing mindfulness. Mindfulness is considered an effective tool for enhancing emotional regulation and mental health (González-Martín et al., 2023). A large body of empirical research shows that individuals with higher levels of mindfulness have better emotional regulation abilities (Xie et al., 2023), stronger psychological resilience (Zhang et al., 2022), and lower perceived stress (Chen et al., 2023). When individuals face overwhelming stress, suicidal thoughts may arise. However, mindfulness indirectly affects suicidal ideation by enhancing vitality (Moscardini et al., 2023).

As an important health-promoting behavior, physical exercise has been widely proven to have a positive effect on mental health. Given that the attentional patterns and attitudes adopted in physical exercise align closely with the core principles of mindfulness, it is important to consider trait mindfulness as a new perspective in research on the psychological effects of physical exercise (Lin, 2023). Current research reveals that exercise can directly improve emotional experiences by stimulating the secretion of neurotransmitters such as endorphins (Zhang et al., 2023; Fuentealba-Urra et al., 2023; Mu et al., 2024). Moreover, the focused state during exercise helps cultivate individual attention and enhances self-efficacy (Lee et al., 2022). It also improves mental health by prolonging exercise duration (Luo et al., 2023). In recent years, some studies have begun to explore the relationship between physical exercise and mindfulness. For example, college students who engage in long-term aerobic exercise exhibit higher levels of trait mindfulness (Yuan et al., 2025), and among students with low levels of mindfulness, the improvement in self-control and sleep quality is more significant (Yin et al., 2024). Based on this evidence, this study proposes that physical exercise can enhance concentration, improve emotional states, and thus increase the trait mindfulness levels of female college students. Therefore, the following hypothesis is proposed:

*H1:* Physical exercise is positively correlated with trait mindfulness in female college students.

When facing smartphone addiction, physical exercise is believed to reduce the risk of smartphone addiction by improving innovative behavior and mental health (Tong and Meng, 2023), and by enhancing self-control, it indirectly influences smartphone addiction tendencies (Wang et al., 2024). Trait mindfulness is also considered an important protective factor in the context of smartphone addiction (Ge et al., 2023). Studies show that mindfulness regulates smartphone addiction by reducing tendencies toward boredom (Regan et al., 2020). Individuals with higher levels of mindfulness have stronger willpower and self-control, thus reducing the likelihood of smartphone addiction (Li et al., 2023). A series of mental health problems caused by smartphone addiction has become an important issue with broad social significance (Li et al., 2022). Therefore, it is urgent to help female college students establish self-regulation mechanisms through psychological intervention, behavioral guidance, and healthy lifestyle patterns in the digital age, achieving a balanced development of physical and mental health.

In the process of physical exercise affecting trait mindfulness, smartphone addiction plays a mediating role, rather than being a direct outcome variable of physical exercise (Arpaci and Kocadag Unver, 2020). Specifically, physical exercise enhances individuals’ self-control ability, reduces smartphone addiction levels, and this change further promotes the improvement of trait mindfulness (Cheng et al., 2024). Therefore, smartphone addiction serves as a mediating variable, not an outcome variable, in this process. Through this mechanism, physical exercise not only directly affects mindfulness levels but also indirectly exerts its influence by regulating smartphone addiction.

Based on this, the following hypothesis is proposed:

*H2:* Smartphone addiction mediates the effect of physical exercise on trait mindfulness.

*H2a:* Physical exercise negatively predicts smartphone addiction in female college students.

*H2b:* Smartphone addiction negatively predicts trait mindfulness in female college students.

Currently, most studies focus on the relationships between physical exercise, smartphone addiction, and trait mindfulness, exploring the relationship between any two of these factors. This study introduces a new methodology by considering smartphone addiction as a mediating variable, revealing the internal interaction between these three factors. This provides valuable guidance for expanding the application of mindfulness theory in sports psychology and behavioral addiction. Research shows that female college students tend to exhibit higher smartphone addiction tendencies compared to male students, especially in terms of emotional regulation and social needs (Pirwani and Szabo, 2024). For instance, some studies have found that females, when emotionally distressed, are more likely to seek instant gratification through smartphones, thus increasing the risk of smartphone addiction (Zhu et al., 2024; Gholamian et al., 2019). This gender difference presents a significant challenge for female college students in dealing with smartphone addiction, providing theoretical support for this study’s hypothesis of smartphone addiction as a mediating variable.

**Figure 1:**
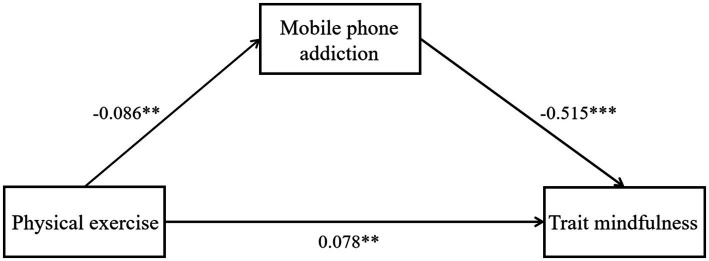
The mediating effect model of mobile phone addiction. This figure illustrates the relationship model between physical exercise, smartphone addiction, and trait mindfulness. The analysis results indicate a significant negative correlation between physical exercise and smartphone addiction (*β* = −0.086, *p* < 0.01), meaning individuals with higher levels of physical exercise tend to show lower levels of smartphone addiction. Additionally, there is a significant negative correlation between smartphone addiction and trait mindfulness (*β* = −0.515, *p* < 0.001), suggesting that individuals with higher levels of smartphone addiction tend to have lower levels of trait mindfulness. Meanwhile, there is a significant positive correlation between physical exercise and trait mindfulness (*β* = 0.078, *p* < 0.01), indicating that individuals with higher levels of physical exercise tend to exhibit higher levels of trait mindfulness. Overall, smartphone addiction plays a mediating role in the influence of physical exercise on trait mindfulness, meaning that physical exercise indirectly enhances trait mindfulness by reducing smartphone addiction tendencies.

Traditional gender concepts still subtly shape the behavioral patterns of women. For instance, the stereotype of “quiet and refined ladies” might cause some female college students to avoid intense physical activities and instead prefer “gentle” ones. The mainstream aesthetic standards often emphasize “thinness as beauty” rather than a healthy physique, leading some female college students to manage their figures through dieting rather than exercise. The image anxiety on social media further amplifies this phenomenon. This study focuses on female college students, exploring the impact of physical exercise on their mindfulness levels and the mediating role of smartphone addiction, using a mediation model (see Figure 1) for exploration. The study also verifies the long-term effects of exercise on emotional health, revealing smartphone addiction as a potential mechanism for behavioral disorders. The research findings can provide evidence for universities to design “physical exercise combined with mindfulness” intervention programs, helping female college students regulate their emotional states and reduce smartphone addiction (Table 1).

**Table 1:** Sample distribution.

University	Number of sampled classes	Nunber of students
Fuzhou University	4	102
Sanming University	6	114
Huaqiao University	8	128
Jimei University	6	105
Minnan Normal University	6	111
Total	30	560

The actual number of returned samples was 554 (response rate 98.93%).

While the proposed model posits physical exercise as a key driver of mindfulness through reduced smartphone addiction, alternative pathways warrant consideration. For instance, pre-existing mental health conditions (e.g., anxiety or depression) may confound the relationship, as they could independently influence both exercise habits and smartphone usage patterns. Similarly, social support networks might serve as a competing mediator, given their documented effects on both mindfulness and digital behaviors. Furthermore, academic pressure may modulate these correlations: during periods of high pressure, students may give up exercising, increase their mobile phone usage as a coping mechanism, and weaken mindfulness, thereby complicating the direction effect we have proposed. Future research should incorporate these variables to isolate the unique contribution of physical exercise. Nevertheless, the current study controls for demographic covariates (e.g., academic major, relationship status) to mitigate baseline confounding (Table 2).

**Table 2:** Demographic differences in physical exercise, mindfulness, and phone addiction.

Variables	*N* (%)	PE		MPA		MIN	
		Mean±SD	t/f	Mean±SD	t/f	Mean±SD	t/f
Total	554	16.04 ± 15.92		47.93 ± 9.77		53.19 ± 11.46	
Residence			−1.95**		0.23		−1.66
Rural	225 (40.6)	14.45 ± 14.62		48.05 ± 9.07		52.81 ± 11.87	
Urban	329 (59.4)	17.13 ± 16.69		47.85 ± 10.23		54.46 ± 11.82	
Only-child status			0.13		4.31***		−1.58
Yes	161 (29.1)	16.18 ± 17.40		50.68 ± 11.13		52.58 ± 12.47	
No	393 (70.9)	15.98 ± 15.30		46.80 ± 8.92		54.28 ± 10.99	
Academic major			4.04***		−0.03		0.44
Science	181 (32.7)	19.73 ± 18.85		47.92 ± 9.49		54.08 ± 11.77	
Liberal arts	373 (25.8)	14.07 ± 13.74		47.94 ± 9.93		53.63 ± 11.30	
Relationship status			−0.71		5.06***		−3.03
Single	411 (74.2)	15.76 ± 15.58		49.15 ± 8.88		52.92 ± 11.39	
Partnered	143 (25.8)	16.85 ± 16.87		44.47 ± 11.29		52.22 ± 11.32	
Categories of sports			0.75		17.15***		21.86***
Mind–body regulation activities	110 (19.9)	17.43 ± 18.26		42.27 ± 8.48		60.25 ± 9.66	
Aerobic exercises	213 (38.4)	16.01 ± 15.65		47.54 ± 8.39		55.23 ± 10.09	
Combat sports	94 (17.0)	15.37 ± 14.05		51.90 ± 10.04		48.00 ± 11.16	
Strength/skill-based activities	63 (11.4)	17.41 ± 16.29		50.05 ± 12.16		51.53 ± 13.31	
Leisure and entertainment sports	74 (13.4)	13.74 ± 14.98		50.64 ± 8.78		49.43 ± 10.65	

PE, physical exercise; MIN, mindfulness; MPA, mobile phone addiction. * *p* < 0.05, ** *p* < 0.01, *** *p* < 0.001.

## 2 Methods

### 2.1 Data collection and sample

This study employed a stratified cluster sampling method, as presented in Table 3, selecting 5 universities in Fujian Province (Fuzhou University, Sanming University, Huaqiao University, Jimei University, and Minnan Normal University). In the stratified cluster design, the stratification was based on grade levels (from freshman to senior), and the cluster units were natural teaching classes. Two classes were randomly selected from each grade for questionnaire surveys. The proportions for the first year were 31%, for the second year 26.9%, for the third year 22.4%, and for the fourth year 19.1%. The average age was 21.09 years (SD = 1.03) (Su and He, 2024).

**Table 3:** Correlation coefficients between the study variables.

Variables	M ± SD	1	2	3	4	5	6	7
Physical exercise	16.04 ± 15.92	1						
Trait mindfulness	53.79 ± 11.45	0.20**	1					
Withdrawal symptoms	18.85 ± 4.11	−0.11*	−0.35**	1				
Highlight behavior	11.02 ± 2.92	−0.18**	−0.46**	0.67**	1			
Social comfort	9.23 ± 2.45	−0.17**	−0.31**	0.53**	0.49**	1		
Mood change	8.83 ± 2.26	−0.12**	−0.40**	0.67**	0.62**	0.43**	1	
Mobile phone addiction	47.93 ± 9.76	−0.17**	−0.45**	0.91**	0.84**	0.72**	0.81**	1

* *p* < 0.05, ** *p* < 0.01, *** *p* < 0.001.

#### 2.1.1 Questionnaire response and validity

The questionnaires were distributed on-site. The participants’ ages ranged from 19 to 25. All participants signed the informed consent form and agreed to have their data anonymized and used solely for academic research. To reduce participant fatigue and comprehension errors, the questionnaire content was arranged in a logical order. The questionnaire design aimed to be concise and clear. Researchers provided guidance and answered questions on-site, while emphasizing the anonymity and voluntary participation principles of the questionnaire. A total of 560 questionnaires. After rigorous verification (including checking for abnormal response patterns, logical contradictions, abnormal filling times, and missing key items), 6 invalid questionnaires were eliminated, and a total of 554 valid questionnaires were obtained. The effective recovery rate was 98.93% (Huang and He, 2020).

#### 2.1.2 Scale standards and sources

This study used the Mobile Phone Addiction Tendency Scale (MPATS), and a score above 48 was used as the criterion for determining smartphone addiction (Wang et al., 2024). The MPATS cutoff (>48) was validated specifically for Chinese college students in the scale’s original development (Xiong et al., 2012), where it showed high sensitivity (82%) and specificity (79%) in distinguishing addictive vs. non-addictive phone use. This cutoff aligns with sociocultural norms of mobile phone use among Chinese undergraduates, supporting its appropriateness here.

#### 2.1.3 Research ethics

This study strictly followed ethical guidelines. All participants signed an informed consent form before completing the questionnaire. During the data collection process, participant information was strictly anonymized to ensure confidentiality, and the data was used only for the analysis of this study. The research team is committed to ensuring the security of the data and complying with ethical standards. The ethics approval for the collection of raw data was granted by the Ethics Review Committee of the Medical School of Huaqiao University (Approval No. M2025004). Detailed descriptions, including the questionnaire and the raw data used in this study, can be accessed in the Supplementary File S1 and Supplementary Table S2.

### 2.2 Measurements

#### 2.2.1 Physical Activity Rating Scale-3

The Physical Activity Rating Scale-3 (PARS-3), the scale was developed by Japanese scholar Kimio Hashimoto and subsequently adapted by Chinese scholar Liang, and has since been widely applied in studies involving Chinese populations (Cao et al., 2024), was used to assess the physical activity levels of the participants. This measurement calculates exercise volume through three aspects: exercise intensity, exercise duration, and exercise frequency. Employing a standardized computational formula: Exercise Volume Index = Intensity × (Duration − 1) × Frequency, it obtains the comprehensive exercise volume indicator, which is in line with our goal of quantifying the overall physical activity of female college students. The scores are classified into 3 categories from low to high: low exercise volume (scores range from 0 to 19); moderate exercise volume (scores range from 20 to 42); high exercise volume (scores range from 43 to 100). It is widely used among Chinese college students, ensuring cultural appropriateness. In this study, the internal consistency reliability (*α* = 0.78) was confirmed.

#### 2.2.2 Mindful Awareness Attention Scale

The Mindful Awareness Attention Scale, developed by Brown and Ryan (2003), was used in its Chinese version (Caycho-Rodríguez et al., 2021). After cross-cultural adaptation, it has been widely cited in mindfulness research among Chinese populations. The scale uses a 6-point Likert scale (1 = strongly agree, 6 = strongly disagree), with a total score of 90, where higher scores indicate higher levels of mindfulness. In this study, the internal consistency reliability (*α* = 0.78) was confirmed.

#### 2.2.3 Mobile Phone Addiction Tendency Scale

The Mobile Phone Addiction Tendency Scale, developed by Chinese scholars Xiong et al. (2012), was used in this study. This scale is divided into four dimensions: salience, social comfort, withdrawal symptoms, and mood changes, assessing mobile phone addiction severity. Higher scores indicate a stronger addiction tendency. The MPATS cutoff (>48) was validated specifically for Chinese college students in the scale’s original development. This cutoff aligns with sociocultural norms of mobile phone use among Chinese undergraduates. Therefore score above 48 suggests possible mobile phone addiction. The scale’s overall Cronbach’s α was 0.89, and the coefficients for its four dimensions were 0.77, 0.74, 0.81, and 0.76 respectively, all within the acceptable range. This confirms the scale’s suitability and reliability for the study.

### 2.3 Data analysis

This study employed IBM SPSS Statistics (version 26.0, IBM Corporation) for data analysis. The commonly used methods included deviation testing, descriptive statistics, and correlation analysis. Before conducting correlation analysis and regression analysis (including mediation analysis), the regression assumptions, such as normality of continuous variables (evaluated through skewness and kurtosis) and multicollinearity (evaluated through variance inflation factor VIF) were tested. The results all met the analysis requirements (Podsakoff et al., 2024). To analyze the mediating effect of mobile phone addiction, this study used Model 4 of the PROCESS macro program developed by Hayes’ team (Falk et al., 2023). Bootstrap resampling method was used to calculate 5,000 resampled data, and the 95% confidence interval of the mediating effect was obtained. Moreover, this 95% confidence interval did not include 0.

## 3 Results

### 3.1 Common method bias testing

A Harman single-factor test was conducted on this data. The results showed that three common factor with eigenvalue >1, and the maximum common factor explained rate was 23.03%, which was lower than the 40% critical standard. This indicates that there was no serious deviation problem in the research data regarding common method bias. Furthermore, confirmatory factor analysis (CFA) was conducted on the common latent factor model. The results showed that there was no significant improvement in model fit (χ^2^/df = 4.023, RMSEA = 0.036, CFI = 0.929, TLI = 0.912, and IFI = 0.891). Overall, the overall model’s fit adequacy is reasonable.

### 3.2 Descriptive statistics of the research variables

The descriptive statistics on physical exercise, trait mindfulness and mobile phone addiction of female college students show that in terms of physical exercise, the average score of female college students is 16.04 points (SD = 15.92), and the overall amount of exercise is relatively low. Among them, 398 students (71.8%) were in a state of low physical activity, 109 students (19.7%) had moderate physical activity, and only 47 students (8.5%) were in a state of high-intensity physical exercise. In terms of trait mindfulness, the average score of the trait mindfulness level of female college students was 53.79 (SD = 11.45), indicating that the mindfulness level of this group was at a medium level, but there were significant individual differences. In terms of mobile phone addiction, the average score of female college students was 47.93 points (SD = 9.76). Among them, 307 female college students (55.4%) had no tendency towards mobile phone addiction, while 247 female college students (44.6%) had a tendency towards mobile phone addiction. Subgroup analysis of exercise types showed significant differences in mindfulness and mobile phone addiction: Mind–body exercises (e.g., yoga, Tai Chi) were associated with the highest mindfulness scores (60.25 ± 9.66) and the lowest mobile phone addiction (42.27 ± 8.48), followed by aerobic exercises (mindfulness: 55.23 ± 10.09; addiction: 47.54 ± 8.39). Team sports (e.g., basketball) had lower mindfulness scores (48.00 ± 11.16) but moderate addiction reduction (51.90 ± 10.04). Statistical tests confirmed these differences (*F* = 21.86, *p* < 0.001 for mindfulness; *F* = 17.15, *p* < 0.001 for addiction), with large effect sizes (η^2^ = 0.13 and 0.11, respectively).

### 3.3 Difference testing of demographic variables with physical exercise, trait mindfulness, and mobile phone addiction

In order to explore the differences in physical exercise, trait concentration ability and mobile phone addiction among different population characteristics (such as grade, place of origin, whether they are the only child, etc.), an independent sample t-test was used to examine the relationship between the population variables and the core research variables, and Cohen’s d was calculated for the significant groups to explain its practical significance. It showed that there were significant differences in physical exercise among students from different places of origin and different majors. Specifically, urban female college students exercised more than their rural counterparts (*p* < 0.01, d = 0.17). Female college students majoring in science had significantly higher addiction to mobile phones than those majoring in liberal arts (*p* < 0.001, d = 0.34). There were significant differences in mobile phone addiction tendency among students from different family statuses and different relationship statuses. Specifically, female college students who were only children had significantly higher addiction tendency to mobile phones than those who were in love (*p* < 0.001, d = 0.40); Female college students in love had significantly lower addiction tendency to mobile phones than those who were single (*p* < 0.001, d = 0.48). A difference analysis of the most frequently participated exercise types in the past 3 months showed that female college students who participated in physical and mental exercise had the lowest score for mobile phone addiction (second was aerobic, strength/technique, leisure, and confrontation), and the highest score for trait mindfulness (second was aerobic, strength/technique, leisure, and confrontation). For sports categories, significant differences in mindfulness (*p* < 0.001) were associated with a medium effect size (Cohen’s *f* = 0.39, η^2^ = 0.13), indicating that sport type explains a substantial portion of variance in mindfulness levels. Detailed information is provided in Appendix Table 2.

### 3.4 Correlation coefficients between the study variables

The Pearson correlation analysis was utilized in this section, with control variables (age, residence, academic major, whether being an only child, and relationship status), to comprehensively explore the associations among the three variables. As presented in Table 3, the results revealed a significantly positive correlation between physical exercise and trait mindfulness among female college students (*p* < 0.01). Additionally, physical exercise exhibited significantly negative correlations with all dimensions of mobile phone addiction (*p* < 0.01). Moreover, all dimensions of mobile phone addiction were significantly negatively correlated with trait mindfulness (*p* < 0.01). This implies that higher levels of trait mindfulness are associated with lower levels of mobile phone addiction and its respective dimensions. Consequently, a higher total amount of physical exercise among female college students is related to greater trait mindfulness and reduced mobile phone addiction across all its dimensions. Risk prediction models for falls.

### 3.5 Mediating effect testing of mobile phone addiction

Based on the previous assumptions, physical exercise was designated as the independent variable and trait mindfulness as the dependent variable. Age, place of residence, academic major, and relationship status were included as covariates, while mobile phone addiction served as a mediating variable. The residuals of all regression models were tested for normality. The results from both statistical tests and graphical inspection showed that the residual distribution largely conformed to the normality assumption. The results of the Process stepwise regression analysis method in Table 4 showed that in Model 1, physical exercise among female college students significantly positively predicted trait mindfulness (*β* = 0.123, t = 4.060, *p* < 0.001), indicating that the overall effect was significant. In the test of Model 2, physical exercise had a significant negative impact on mobile phone addiction (*β* = −0.086, t = −3.320, *p* < 0.01). After including the mediating variable, mobile phone addiction in the third model, the predictive effect of physical exercise on trait mindfulness remained significant (*β* = 0.078, t = 2.861, *p* < 0.01). In addition, mobile phone addiction can significantly negatively predict trait mindfulness (*β* = −0.515, t = −11.546, *p* < 0.001). Specifically, for every 1-point increase in physical exercise, mindfulness levels increase by 0.123 points; at the same time, smartphone addiction levels decrease by 0.086 points. For every 1-point decrease in smartphone addiction levels, mindfulness levels increase by 0.515 points. In other words, exercise reduces smartphone addiction and further increases mindfulness levels. According to the mediation effect test criteria proposed by Hayes (2013), mobile phone addiction plays a mediating role between physical exercise and the influence of trait mindfulness on female college students (Table 5).

**Table 4:** Test of mediating effect by stepwise regression analysis method.

Outcome variable	Predictor variable	R	R-sq	F	*β*	t
MIN	PE	0.170	0.029	16.486***	0.123	4.060***
MPA	PE	0.140	0.020	11.021**	−0.086	−3.320**
MIN	PE	0.467	0.218	76.868***	0.078	2.861**
	MPA				−0.515	−11.546***

* *p* < 0.05, ** *p* < 0.01, *** *p* < 0.001.

**Table 5:** Bootstarp analysis of the mediating effect.

Effect	Path	Effect value	BootSE	BootLLCI	BootULCI	Relative mediation effect (%)
Total indirect effect	PE → MIN	0.123	0.030	0.063	0.182	
Direct effect	PE → MIN	0.078	0.027	0.025	0.132	63.41%
Indirect effect	PE → MPA → MIN	0.044	0.014	0.019	0.073	35.77%

* *p* < 0.05, ** *p* < 0.01, *** *p* < 0.001.

Bootstrap analysis was used to examine the mediating role of mobile phone addiction. The results show that the direct path effect value of physical exercise → trait mindfulness is 0.078, and its 95% confidence interval [0.025, 0.132] does not include 0, indicating that this path effect is significant, that is, physical exercise can directly affect the trait mindfulness level of female college students. The effect size of the indirect path of physical exercise → mobile phone addiction → trait mindfulness is 0.044, and its 95% confidence interval [0.019, 0.073] does not include 0, indicating that this path is also significant. This indicates that physical exercise can directly improve the trait mindfulness level of female college students. It can also have an indirect impact on their trait mindfulness by alleviating their mobile phone addiction tendency. The mediating effect of mobile phone addiction accounts for 35.77% of the total effect and plays a partial mediating role.

**Figure 2:**
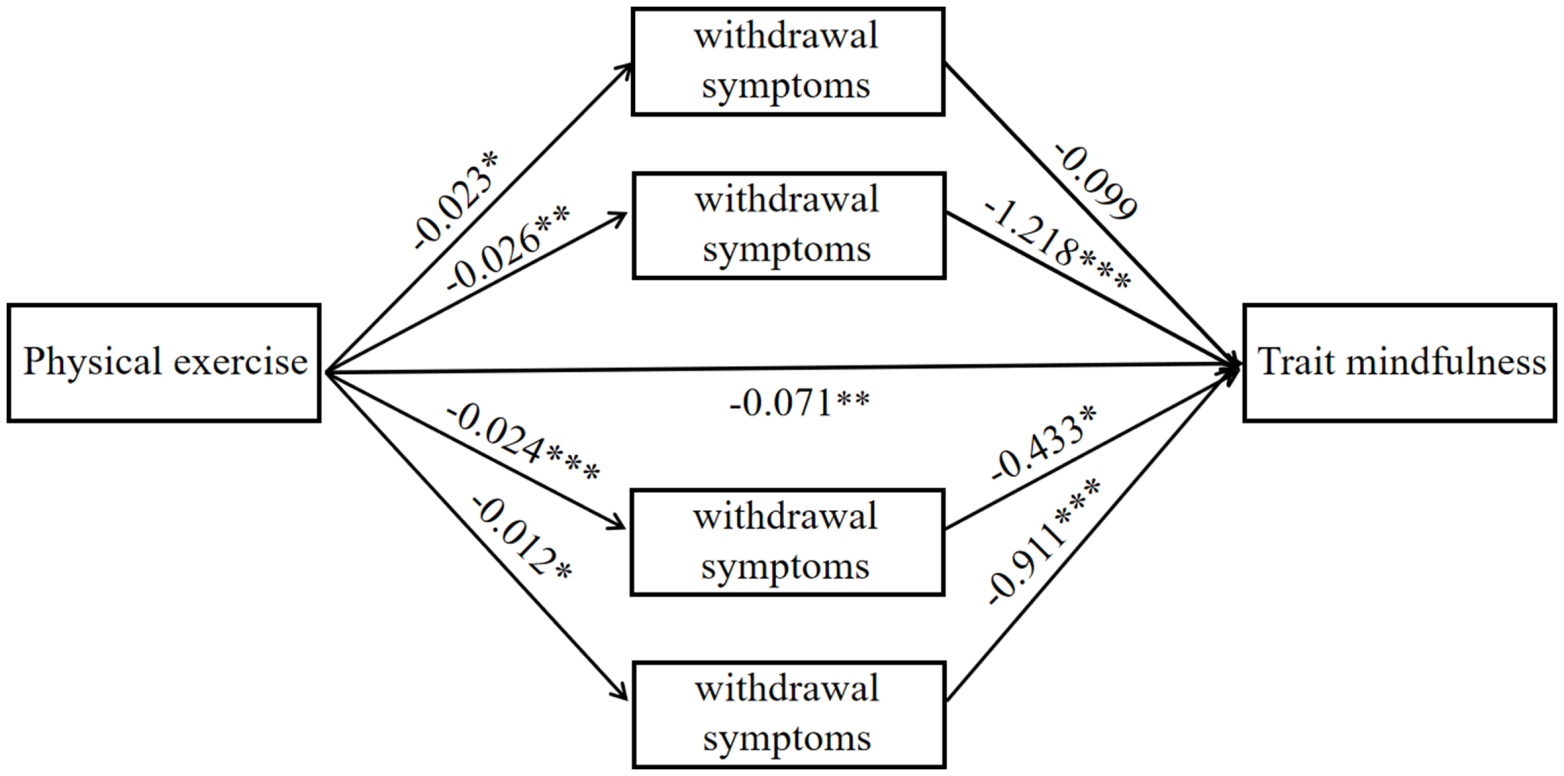
Mediating effect model of mobile phone addiction in various dimensions. This figure presents the relationship model between physical exercise, withdrawal symptoms, and trait mindfulness. The analysis results show a significant negative correlation between physical exercise and withdrawal symptoms (*β* = −0.023, *p* < 0.05), indicating that individuals with higher levels of physical exercise show fewer withdrawal symptoms. Furthermore, withdrawal symptoms have a significant negative impact on trait mindfulness (*β* = −0.433, *p* < 0.01), meaning the more severe the withdrawal symptoms, the lower the individual’s trait mindfulness. Further analysis reveals that physical exercise indirectly promotes the enhancement of trait mindfulness by reducing withdrawal symptoms (*β* = −0.026, *p* < 0.01). In addition, different dimensions of withdrawal symptoms have varying degrees of impact on trait mindfulness, especially certain withdrawal symptoms (*β* = −1.218, *p* < 0.001), which have a significantly stronger negative effect on trait mindfulness. Overall, withdrawal symptoms play a significant mediating role between physical exercise and trait mindfulness, particularly in the process of alleviating withdrawal symptoms, where physical exercise indirectly strengthens an individual’s trait mindfulness.

The four dimensions of the mobile phone addiction questionnaire were incorporated into the mediating effect model for a more specific analysis (see Table 6). The results showed that the mediating effect of withdrawal symptoms between physical exercise and trait mindfulness was not statistically significant. The partial mediating effects of highlighting behavior, social comfort, and mood change are statistically significant. Among them, the effect value of highlighting behavior is the highest, at 0.032, the mediating effect value of social comfort is 0.010, and that of mood change is 0.011. The mediating effect proportions of the three are 18.70, 8.13, and 8.94%, respectively. Based on the above results, a mediating effect model diagram was constructed (Figure 2).

**Table 6:** Analysis of the mediating effect of mobile phone addiction in various dimensions.

Effect	Path	Effect value	BootSE	BootLLCI	BootULCI	Relative mediation effect (%)
Total indirect effect	PE → MIN	0.123	0.030	0.063	0.182	
Direct effect	PE → MIN	0.071	0.027	0.009	0.018	57.77%
Indirect effect	PE → WS → MIN	0.002	0.015	−0.012	0.005	–
	PE → SB → MIN	0.032	0.004	0.013	0.055	18.70%
	PE → SS → MIN	0.010	0.011	0.000	0.024	8.13%
	PE → MC → MIN	0.011	0.006	0.001	0.026	8.94%

WS, withdrawal symptoms; SB, salient behavior; SS, social soothing; MC, mood change; * *p* < 0.05, ** *p* < 0.01, *** *p* < 0.001.

## 4 Discussion

The relationship between physical exercise and mindfulness level in female college students is significant, as the results indicate that physical exercise can significantly enhance the trait mindfulness level of female college students (*β* = 0.123, *p* < 0.001), verifying *H1*. This finding supports the positive role of physical exercise in promoting mental health, which is consistent with previous studies. Physical exercise can influence the psychological quality of college students by improving their mindfulness levels (Davis and Clark, 2019). The positive impact of physical exercise on the mindfulness level of female college students may have multiple mechanisms.

From the perspective of Exercise Psychology, the concentration state during exercise is highly consistent with “attention control” in mindfulness. During long-term regular physical exercise, it can help individuals develop the ability to observe the current experience, which is consistent with the characteristics of mindfulness such as “non-judgment” and “acceptance” (Davis and Clark, 2019). From the perspective of neurobiology, physical exercise can stimulate the secretion of various neurotransmitters such as endorphins, dopamine, etc., and increase the concentration of brain-derived neurotrophic factor (BDNF) (Curtis et al., 2024; Muñoz Ospina and Cadavid-Ruiz, 2024). Endorphins can induce a sense of pleasure, relieve stress and anxiety, and regulate neural activity in the brain, thereby enhancing self-awareness and impulse control (Sharma and Sharma, 2020). Dopamine is closely related to cognitive functions such as attention (Smallwood and Schooler, 2015), memory, and fear (Riem et al., 2012; Gonzalez et al., 2013). The increase in its secretion helps maintain a high level of concentration. Based on the aforementioned neurobiological correlations and existing literature, a speculative explanation can be made: Female college students are more likely to enter a state of concentration and calmness after physical exercise, enhancing their perception of the current emotions, reducing their tendency towards mobile phone addiction, and improving their mindfulness levels. From the perspective of psychological perspective, physical exercise provides a context for female college students to focus on bodily activities, and can be regarded as “moving mindfulness training.” For example, the differential effects of exercise types on mindfulness and mobile phone addiction can be attributed to their distinct attentional demand characteristics. Types of movement that emphasize internal perception (such as Tai Chi and yoga) are more likely to promote the improvement of mindfulness (Muzik and Diwadkar, 2019). Such movements require attention to be concentrated on breathing rhythms and body sensations, which can effectively cultivate concentration and awareness, reducing distraction and distractions. Aerobic exercises (e.g., jogging) involve sustained attention to physiological states, such as heart rate and pace, promoting continuous engagement in the present moment, which in turn enhances mindfulness. In contrast, team sports (such as basketball and football) emphasize social interaction and strategic coordination, directing attention outward. Although they may reduce addiction by fulfilling social needs, their limited focus on internal awareness constrains their effectiveness in cultivating mindfulness. These findings suggest the potential for tailored interventions: mind–body and aerobic exercises may be particularly effective for enhancing mindfulness, whereas team sports could serve as complementary options for addressing addiction through social engagement. Studies have shown that there is an interaction between physiological and psychological processes. Behaviors combining the effects of stimulation and meditation techniques not only affect the homeostatic function and immune response, but also the endogenous release of endocannabinoids and endorphins at the molecular level, which has particularly obvious effects on improving emotions (You and Liu, 2022). Sala et al. conducted a randomized pilot study and found that participants who engaged in a mindfulness-based physical activity intervention exhibited greater improvements in emotional regulation and stress reduction compared to those who practiced either mindfulness or physical activity alone. This synergistic effect is attributed to the integration of physical movement, which enhances attentional focus through rhythmic engagement, and mindfulness, which fosters non-judgmental awareness of present experiences (Sala et al., 2021). Similarly, Balciuniene et al. demonstrated that an education and mindfulness-based physical activity program significantly promoted positive body image and reduced anxiety among female students, outperforming single-modality interventions (Balciuniene et al., 2021). Additionally, Fabian et al. highlighted that combined interventions (though focusing on cognitive stimulation and omega-3 fatty acids alongside exercise) can induce neuroplastic changes in brain regions associated with emotional regulation, providing a neurobiological basis for the superior efficacy of combined approaches in improving mental health outcomes, including reducing addictive behaviors like smartphone overuse (Köbe et al., 2016). Therefore, physical exercise may promote the improvement of female college students’ mindfulness levels through a series of reactions, helping them to face various stressors and negative emotions more calmly in daily life.

The mediation effect analysis indicates that mobile phone addiction plays a mediating role in the relationship between physical exercise and the positive mindfulness of college students. Specifically, physical exercise can negatively predict female college students’ mobile phone addiction (*H2a*), and mobile phone addiction can negatively predict female college students’ positive mindfulness (*H2b*). Thus, *H 2* is supported. The indirect effect of mobile phone addiction on physical exercise and trait mindfulness was statistically significant (*β* = 0.078, 95% CI does not include 0). It should be noted that this effect value is within a relatively small range, suggesting that the actual influence intensity of this mediating path in the current sample is limited. From a practical perspective, this small effect may be related to the characteristics of the research group, as mentioned earlier, 71.8% of the participants had a low level of physical activity, and limited behavioral variations may have compressed the manifestation space of the effect. Nevertheless, this result still provides preliminary clues for understanding the relationship among the three. Clinically, this suggests that exercise-based mindfulness interventions may need to combine reducing smartphone addiction with other strategies (such as mindfulness training) to achieve meaningful change. Future research should focus on identifying the conditions under which effect sizes may increase, such as in populations with higher levels of addiction, to enhance the clinical utility of these findings. Among the various dimensions of mobile phone addiction, the mediating effect of withdrawal symptoms (WS) is not significant, contrasting with the significant mediating roles of highlighting behavior (SB), social comfort (SS), and emotional changes (MC). The 18.70% mediating proportion of salient behavior suggests that interventions targeting the “attention occupation” in mobile phone use (e.g., attention training during exercise) may be an efficient entry point for improving mindfulness. Meanwhile, the effects of mood change (8.94%) and social comfort (8.13%) indicate that exercise’s promotion of emotional regulation and real-world social interaction, though indirect, is indispensable and can serve as auxiliary intervention directions. This difference may stem from the unique nature of withdrawal symptoms (such as anxiety and restlessness when not using the phone), which are related to neuroadaptation. The brain has become accustomed to continuous phone stimulation, and when phone use is interrupted, it causes physical discomfort. Although physical exercise helps with psychological self-regulation, its direct impact on this neurobiological dependence is limited, as it cannot directly counteract the physiological cravings associated with withdrawal. Instead, highlighting behavior (placing the phone above daily activities), social comfort (using the phone to alleviate loneliness), and emotional changes (relying on the phone to regulate emotions) are mainly psychological aspects. Physical exercise acts on these dimensions in the following ways: (1) By enhancing concentration during exercise to reduce the bias towards the phone; (2) By providing face-to-face interaction in group exercise to meet social needs (reducing reliance on phone socialization); (3) By releasing endorphins to improve emotional regulation (lowering the need to manage emotions through the phone). These psychological mechanisms are more susceptible to the influence of physical exercise. Female college students who actively participate in physical exercise reduce their reliance on mobile phones due to the fact that exercise occupies a significant amount of time and energy. The social interaction and teamwork experiences brought by physical exercise meet the social needs of female college students, preventing them from overly relying on mobile phones to seek social satisfaction. The study found that positive mindfulness can alleviate mobile phone addiction by reducing social anxiety (You and Liu, 2022). Mobile phone addiction has a negative impact on the positive mindfulness level of female college students (Sala et al., 2021). Long-term addiction to mobile phones can lead to distraction and fragmented thinking, making it difficult for female college students to focus on perceiving their current emotions and environmental changes, thereby reducing their mindfulness level (Balciuniene et al., 2021; Köbe et al., 2016). Mobile phone addiction may also cause social isolation, making female college students neglect interpersonal communication and emotional experiences in real life, further weakening their ability to observe themselves and the surrounding world (Martin and Ergas, 2016). To address this, implementing combined exercise and mindfulness programs in universities is somewhat feasible: universities generally value students’ well-being, with existing wellness centers able to run activities using their resources; students have an inherent interest in mind–body exercises, and digital tools facilitate large-scale promotion (Remskar et al., 2024). However, challenges remain, such as students’ tight schedules, instructors needing expertise in both exercise science and mindfulness, and some students’ cognitive biases toward mindfulness (Martínez-Rubio et al., 2021; Kuyken et al., 2022). These can be tackled by offering short modular courses, strengthening teacher training, linking programs to academic improvement, piloting first in specific groups before gradual promotion, and using incentives (Moore et al., 2020).

Despite the valuable insights, several limitations should be recognized. In this study, the physical exercise of female college students was mostly in a low-intensity state, and the sample size and scope were not extensive enough. The sampling scope and demographic specificity have limited the generalizability of the research results. This situation limits the potential benefits of physical exercise in enhancing the level of mindfulness. Low-intensity physical exercise may not fully stimulate the secretion of neurotransmitters and is difficult to provide sufficient opportunities for concentration training and the improvement of self-efficacy (Basso and Suzuki, 2017). This study found through cross-sectional data that physical exercise can indirectly improve trait mindfulness by reducing mobile phone addiction, providing correlational evidence for understanding the relationship between the three variables. However, it should be clearly stated that the cross-sectional design can only reflect the co-occurrence of variables and cannot determine the causal direction of the “physical exercise → mobile phone addiction → trait mindfulness” pathway. For example, there may be reverse paths (e.g., individuals with high mindfulness are more likely to persist in exercise and reduce mobile phone use) or all three variables may be influenced by common underlying factors (e.g., self-discipline). This design limitation makes the current conclusions more suitable for explaining “correlational patterns” rather than “causal mechanisms.” Furthermore, as a self-reporting scale, it may pose the risk of reporting bias when assessing physical exercise, mobile phone addiction (Zhou and Feng, 2025), and mindfulness levels, including social expectation bias (where participants may report higher addiction tendencies to conform to social expectations) and recall bias (where participants may not accurately recall their mobile phone usage behaviors). These factors may affect the accuracy of the measurement results. Although the PARS-3 scale is widely used among Chinese college students and has good cultural adaptability, it may not cover all dimensions of physical activity, such as the type and environment of exercise, which are not taken into account. The study also lacks exploration of the psychological and social environmental factors that may affect mindfulness or mobile phone addiction. For instance, individual traits such as self-control ability and emotional regulation strategies may interact with the level of mindfulness, thereby influencing mobile phone usage behavior. Additionally, family parenting styles, mobile phone usage norms of peer groups, and the accessibility of sports facilities in universities, all of which may indirectly shape behavioral habits and thereby affect the interrelationship among the three. In addition, mobile phone addiction is not an isolated digital behavior; it may have complex interactions with other digital behaviors (such as social media use, online learning, and e-reading). Moderate social media interaction may alleviate feelings of loneliness and indirectly reduce compensatory use of mobile phones, while excessive reliance on online learning tools may blur the boundaries between work and leisure, thereby exacerbating the tendency towards addiction (Wang et al., 2025). These unconsidered factors suggest that the current model still has limitations in explaining the relationships among variables.

Based on this, future research can be improved in several aspects: conducting longitudinal studies with multiple time points to track the dynamic relationships between physical exercise, mobile phone addiction, and mindfulness, so as to clarify the temporal sequence and the long-term impact of changes in exercise habits on outcomes; implementing randomized controlled trials to compare the effects of different exercise intensities, types and durations while controlling for mindfulness training components, so as to clarify causal mechanisms and dose–response effects; Multivariate analysis was used to statistically control for interrelated demographic factors, thereby more accurately identifying which specific subgroup characteristics were independently associated with exercise, smartphone addiction, or mindfulness. Incorporating objective measures such as accelerometer-based physical activity monitoring and smartphone usage tracking (e.g., screen time logs) to reduce self-report biases and improve the precision of variable assessment; include variables such as emotional regulation, social support, and sleep quality. This approach will better capture the multifaceted nature of the impact of physical exercise on mindfulness, rather than being limited to the single mediating variable examined in this study. Expanding the sample scope to include males, clinical populations (e.g., individuals with diagnosed smartphone addiction), and cross-cultural cohorts, so as to enhance the generalizability of the research results.

## 5 Conclusion

Through empirical analysis, this study demonstrated that the total physical exercise of most female college students is at a low state, and the phenomenon of cell phone addiction is more common. This study concludes that physical exercise has a significant positive effect on the level of trait mindfulness of female college students, and can reduce the tendency of mobile phone addiction through physical exercise, which in turn enhances the trait mindfulness of female college students. This finding enriches the intersection of physical psychology and positive thinking research and provides an actionable and integrated strategy for mental health interventions in universities. Given the overlapping effects of mindfulness training and physical exercise in reducing impulsive smartphone use and enhancing present-moment focus, combining the two in an intervention makes theoretical sense. Considering the complex relationship between digital media use and emotional well-being, schools should pay closer attention to students’ smartphone usage behaviors, encourage them to actively participate in physical exercise, and incorporate “exercise + digital literacy” into their mental health support systems. Schools should offer 1–2 credit hours of “mindfulness exercise” courses each semester. Additionally, schools should provide guidelines for managing phone use (such as phone-free zones in classrooms and a “digital detox” initiative 1 hour before bedtime), reducing triggers for excessive phone use through environmental design. This will help female college students maintain good physical and mental states in the fast-paced online world and high-pressure environment.

## Data Availability

The original contributions presented in the study are included in the article/Supplementary material, further inquiries can be directed to the corresponding author.
