# Supplementary material for: Effects of physical exercise on mindfulness level among female college students: the mediating effect of mobile phone addiction
Source: Front Psychol. 2025 Sep 12;16:1667868. doi: 10.3389/fpsyg.2025.1667868 (PMC12463849; doi:10.3389/fpsyg.2025.1667868)
Supplement: Supplementary file 2 [file Data_Sheet_1.PDF]

S1. Methods supplement

1.1《Basic Information Questionnaire》

1. What is your age?

   ____

2. What academic year are you in?

☐Freshman ☐Sophomore ☐Junior ☐Senior

3. Are you from an urban or rural area?

☐Urban ☐Rural

4. Are you an only child?

☐ Yes ☐No

5. Does your major belong to the liberal arts or sciences category?

☐Liberal arts ☐Sciences

6. Are you single or partnered?

☐Single ☐Partnered

7. Which was the most frequently participated in physical activity by you in the past three months?

☐Mind-body regulation activities (e.g., yoga, Tai Chi, Qigong)

☐Aerobic exercises (e.g., brisk walking, jogging, cycling, swimming)

☐Combat sports (e.g., basketball, football, badminton, martial arts)

☐Strength/skill-based activities (e.g., weightlifting, dance, gymnastics)

☐Leisure and entertainment sports (e.g., hiking, recreational walking, table tennis)

Note: If you participate in multiple activities, please select the one you engage in most regularly.

1.2《Physical Activity Rating Scale-3》

1. What is the intensity of your exercise?

☐Light exercise (such as walking, doing radio calisthenics, etc.).

☐Low - intensity and not very strenuous exercise (such as recreational volleyball, yoga, jogging, Taijiquan, etc.).

☐Moderate - intensity, relatively intense and lasting exercise (such as cycling, running, table tennis, weight - lifting in the gym, etc.).

☐High - intensity exercise that makes you breathe rapidly and sweat a lot, but not lasting (such as badminton, basketball, tennis, football, etc.).

☐High - intensity and lasting exercise that makes you breathe rapidly and sweat a lot (such as sprinting, a set of aerobics, swimming, etc.).

2. How many minutes do you exercise each time at the above - mentioned intensity?

☐Less than 10 minutes.

☐11 to 20 minutes.

☐21 to 30 minutes.

☐31 to 59 minutes.

☐More than 60 minutes.

3. How many times do you engage in the aforementioned activities in a month?
☐Less than 1 time a month.

☐3 to 5 times a week.

☐2 to 3 times a month.

☐Approximately 1 time a day.

☐1 to 2 times a week.

1.3《Mindful Awareness Attention Scale》

	Almost Always	Frequent -ly	Occasion -ally	Seldom	Rarely	Almost Never
1. I find myself preoccupied with the future or the past.						
2. I find myself doing things without paying attention.						
3. I snack without being aware that I'm eating.						
4. I find myself listening to others with half an ear while doing something else at the same time.						
5. I drive on "autopilot" and then wonder why I ended up there.						
6. I find it difficult to focus on what is happening in the present moment.						
7. I tend to walk quickly to get somewhere without noticing what I experience along the way.						
8. I find myself preoccupied with worries about the future or regrets about the past.						

9. I find myself doing things without being fully aware of my actions.						
10. I find it hard to stay focused on my breathing during meditation or relaxation.						
11. I often find myself "spacing out" or daydreaming.						
12. I find it difficult to focus on my physical sensations, such as hunger or fatigue.						
13. I find myself rushing through activities without really noticing them.						
14. I find it hard to stay focused on the present moment when I’m upset.						
15. I find myself doing things automatically, without being conscious of what I’m doing.						

1.4《Mobile Phone Addiction Tendency Scale》

	Strongly Disagree	Disagree	Neutral	Agree	Strongly Agree
1. If I haven’t had my phone with me for a while, I will immediately check for text messages or missed calls.					
2. I prefer chatting via my phone over face-to-face communication					
3. When waiting for someone, I always frequently call them to ask where they are; if I don’t, I feel anxious and restless.					
4. I feel uncomfortable if I haven’t used my phone for a long time.					
5. In class, I cannot concentrate on listening because of phone calls or text messages.					
6. I would feel lonely without a mobile phone.					
7. I feel more confident when communicating with others via my phone.					

8. If my phone doesn’t ring for a while, I feel uncomfortable and subconsciously check for missed calls or text messages.					
9. I often have the illusion that "my phone is ringing/vibrating."					
10. I feel my life is more fulfilling when I receive many calls and text messages.					
11. I often worry that my phone will automatically shut down.					
12. The mobile phone is part of me; if its use is reduced, I feel like I have lost something.					
13. Classmates and friends say I rely too much on my phone.					
14. When my phone frequently fails to connect to the internet or receive signals, I feel anxious and become irritable.					
15. In class, I often take the initiative to focus on my phone, which affects my listening.					
16.I feel more comfortable communicating with others via my phone.					
